# Morphometric study applied to testicular and epididymis hydatids torsion

**DOI:** 10.1038/s41598-024-52734-9

**Published:** 2024-02-08

**Authors:** Renato G. Barbosa, Luciano Alves Favorito, Francisco J. B. Sampaio

**Affiliations:** Urogenital Research Unit, Department of Anatomy, State University of Rio de Janeiro – UERJ, Rua Professor Gabizo, 104/201, Tijuca, Rio de Janeiro, RJ CEP: 20271-320 Brazil

**Keywords:** Translational research, Anatomy, Medical research

## Abstract

Twisted testicular appendages had difficult differential diagnosis with testicular torsion. The objective of this paper is to evaluate the number, shape, size and determine the laterality pattern of the testicular and epididymal hydatids and evaluate the correlations between the length and width of the testicular and epididymal hydatids with testicular measurements. We analyzed 60 fixed cadavers and 16 patients with prostate cancer without previous hormonal treatment undergoing bilateral orchiectomy, totalizing 76 units and 152 testicles. In relation to the testicular appendices, we analyzed the following situations: absence of testicular and epididymis appendages, presence of a testicular appendix, presence of epididymis appendix, and presence of testicular and epididymis appendix. We measured the length, width and thickness of the testis and classified the appendages as sessile or pedicled. Chi-square test was used to verify associations between categorical variables. McNemar Test was used to verify differences between the percentages of right and left appendages. Correlations between quantitative measures were evaluated using the Pearson Correlation Coefficient (*p* < 0.05). In 50 cases (65.78%) we observed the presence of some type of appendices, in 34 cases (44.72%) we observed the presence of testicular appendices and in 19 cases (25%) the presence of epididymal appendices. We observed the presence of pedicled appendices in 39 cases (51.32%), with 25 of the cases (32.89%) of pedicled testicular appendices and 14 of the cases (18.42%) of pedicled epididymal appendages, with a significant association between the occurrence of appendices on the right and left sides (*p* < 0.001). Testicular hydatids were present in around two thirds of our sample being pedunculated in almost half of the cases with bilateral similarity. There is a significant chance in cases of twisted appendices that the same anatomical characteristics are present on the opposite side, which is a factor that tends to indicate the need for contralateral surgical exploration in cases of torsion, however studies with larger samples are needed to confirm these findings.

## Introduction

Testicular torsion (TT) is a urologic urgency, with high incidence in teenagers and young adults^1^. Testicular functional tissue is damaged in many pathways that lead to hypoxia and cellular death and testicular atrophy with irreversible damage if the torsion is not resolved within up to 6 h^2^. Testicular torsion can occur at any age; however, it is more frequent in teenagers and young adults^3,4^. This pathology is responsible for approximately 90% of acute testicular pain in patients between 13 and 21 years old^1,5,6^. Some conditions that do not require surgical exploration can be confused with testicular torsion, being one of the most common the torsion of testicular and epididymis hydatids^5,6^.

Testicular hydatids (appendices) have been considered congenital anomalies in the past^7^, however some studies report that these structures are present in most normal individuals^8,9^. Testicular hydatids varies in size from 1 to 10 mm in diameter^7,8^. If the appendices are long and pedunculated, they can twist on their own axis, causing very painful symptoms and simulating testicular torsion^10,11^.

Torsion of the testicular appendices is amenable to clinical treatment, but the differential diagnosis with testicular torsion is difficult and the patient may be taken to urgent surgical exploration^9,12^. Studies demonstrating whether twisted testicular and epididymal appendices have similar anatomical features on the non-twisted side are rare^13^. Our hypothesis is that the testicular and epididymal appendices present bilateral anatomical similarity.

The objective of the present study is to evaluate the number, shape, size and to determine the laterality pattern of the testicular and epididymal hydatids in human cadavers and in patients with prostate cancer who underwent bilateral orchiectomy. We will also evaluate the correlations between the length and width of the testicular and epididymal hydatids with testicular measurements.

## Material and methods

The study was approved according to the ethical standards of the hospital's institutional committee on experimentation with human beings (IRB:73418622.0.0000.5259). The study has also been registered in the Brazil Plataform, Ministry of Health, National Health Council, National Research Ethics Commission for studies with human beings. We confirm that all methods used in this paper were carried out in accordance with relevant guidelines and regulation in compliance to the declaration of Helsinki.

### Patient population

The sample size was calculated using the simple random sampling formula to estimate a population mean. Standard deviation and absolute error values were used to measure the right and left testicular appendages (length, width, and thickness). According to the results, in order to obtain an estimate with a maximum error of 10% (of the mean value), a minimum sample of 254 and a maximum of 562 cases (appendices) would be required. For an estimate with a maximum error of 15% (of the mean value), a minimum sample of 113 and a maximum of 250 cases (appendices) would be required. On the other hand, for an estimate with a maximum error of 20% (of the mean value), a minimum sample of 64 and a maximum of 140 cases (appendices) would be required.

During the period from July 2022 to July 2023, we studied 60 fixed cadavers and 16 patients with prostate cancer without previous hormonal treatment undergoing bilateral orchiectomy to control the disease, totalizing 76 units and 152 testicles. Cadavers with incisions in the inguinal region and scrotum and patients with previous testicular surgery or inguinal hernia were excluded from the study. Single testicle cases were also excluded. In both cadavers and patients, an incision was made in the median raphe of the scrotum with stratigraphic dissection to access the testicles and identify the appendices.

### Surgical technique

All operations of the patients with prostate cancer were performed using a longitudinal scrotal incisions in median scrotal raphe. We dissected the testicular tunics to access the testis and epididymes. Before the orchiectomy, we performed testicular measurements and noted the presence or absence of testicular hydatids. Following the orchiectomy, the spermatic cord stump was ligated with two 2–0 cotton hemostatic sutures. The tunica dartos was closed with a running colorless Vicryl 4–0 suture and the skin was closed with separate nylon 4–0 stitches.

**Figure 1:**
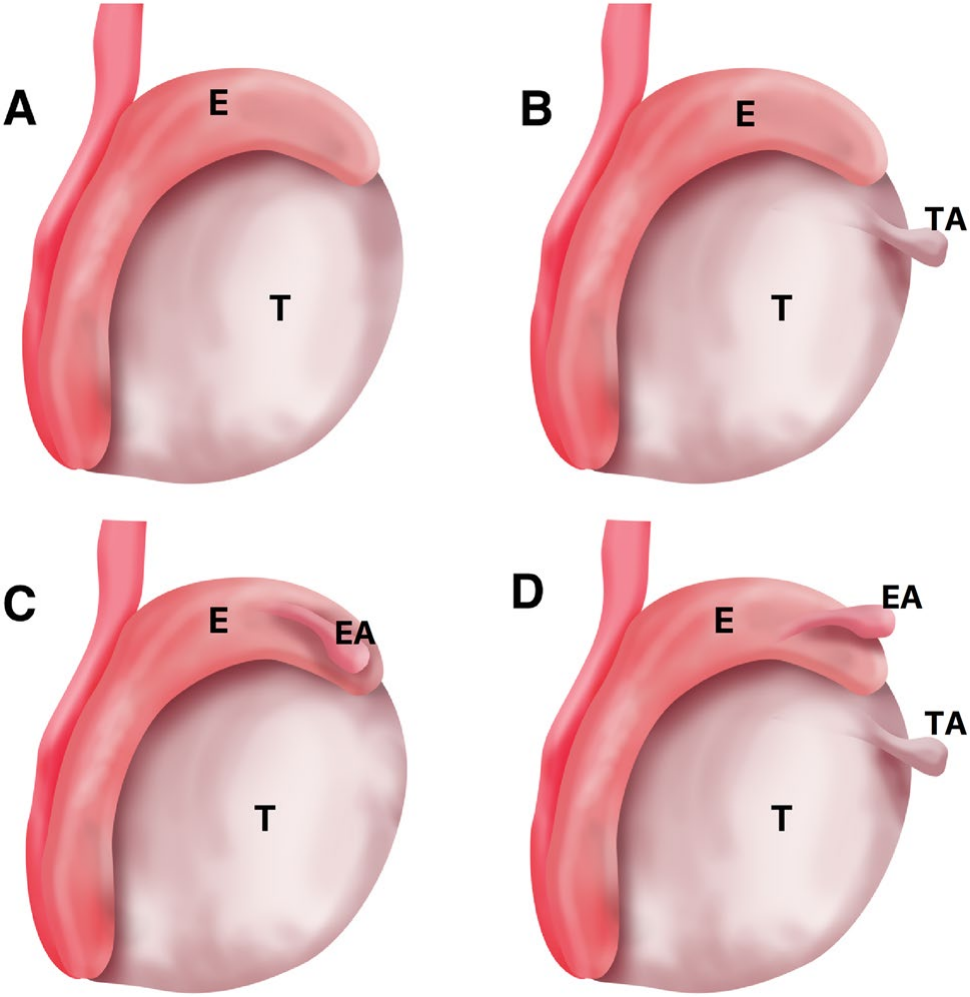
The figure shows a schematic drawing with the types of possible dispositions of the para-testicular apppendix found during the testicular dissections in our sample: (**A**) absence of testicular and epididymis appendix, (**B**) presence of testicular appendix (TA), (**C**) presence of epididymal appendix (EA) and (**D**) presence of testicular and epididymis appendix; T-Testis and E-Epididymis.

In relation to the testicular appendices, we analyzed four situations: absence of testicular and epididymis appendices, presence of a testicular appendix, presence of a epididymal appendix, and presence of both testicular and epididymis appendices (Fig. 1).

**Figure 2:**
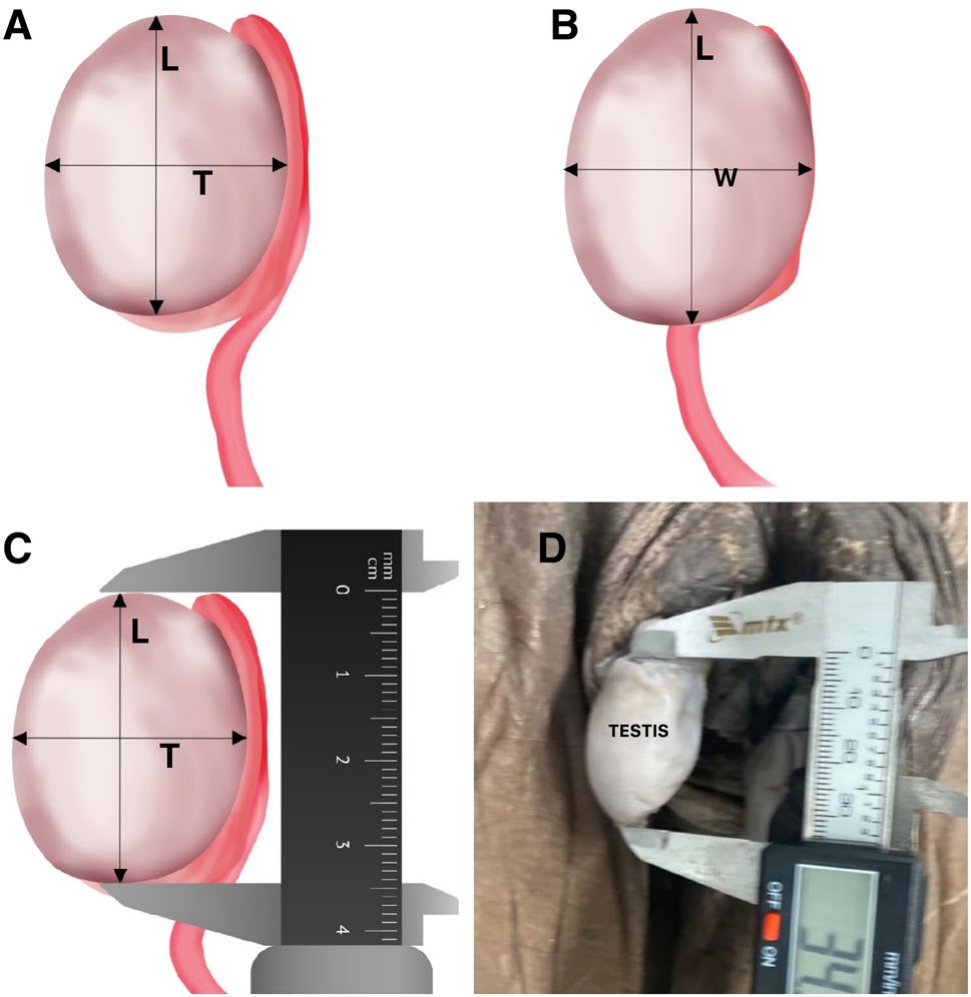
The figure shows schematic drawings and dissection during the measurements of the testicular volume. (**A**) Schematic drawing showing the length (L) and thickness (T) of the testis; (**B**) Schematic drawing showing the length and width (W) of the testis; (**C**) Schematic drawing showing the measurement of testicular length with a digital pachymeter and (**D**) Measurement of testicular length with a digtital pachymeter during the cadaveric dissection.

After the dissection, the testis and appendices were photographed with a digital camera (DP70, Olympus America, Inc., Melville, New York) under the same conditions (same focal distance by the same examiner) at a resolution of 2040 pixels and stored in a TIFF file. With the aid of a digital pachymeter we measured the length, width and thickness of the testis in centimeters (cm). The testicular volume was calculated using the ellipsoid formula: Testicular volume (TV) = [length × thickness × width] × 0.71 is the most used and accurate formula for calculating the testicular volume^14–16^ (Fig. 2).

With the Image J software, version 1.46r, we classified the appendices as sessile or pedicled and measured the length and width of the appendices^17,18^. The measurements were performed by the same observer^19,20^. All measurements were performed, considering the major axis between initial and final points. Data of appendices are expressed in millimeters and the data of the testis are expressed in centimeters.

### Statistical analysis

All parameters were statistically processed and graphically described. The Chi-square test was used to verify associations between categorical variables. The McNemar Test was used to verify differences between the percentages of right and left appendages. Correlations between quantitative measures were evaluated using the Pearson Correlation Coefficient. A significance level of 5% (*p* < 0.05) was used in all tests. Simple linear correlations (where r^2^ values less than 0.4 reflect very weak correlation, r^2^ between 0.4 and 0.7 reflect moderate correlation and r^2^ greater than 0.7 indicate strong correlation) were calculated for some quantitative variables. The statistical analysis was performed with the IBM SPSS program version 20.

### Ethical approval

The study was approved according to the ethical standards of the hospital's institutional committee on experimentation with human beings (IRB:73418622.0.0000.5259).

### Informed consent

We confirm that informed consent was obtained from all subjects of the patients studied.

## Results

**Figure 3:**
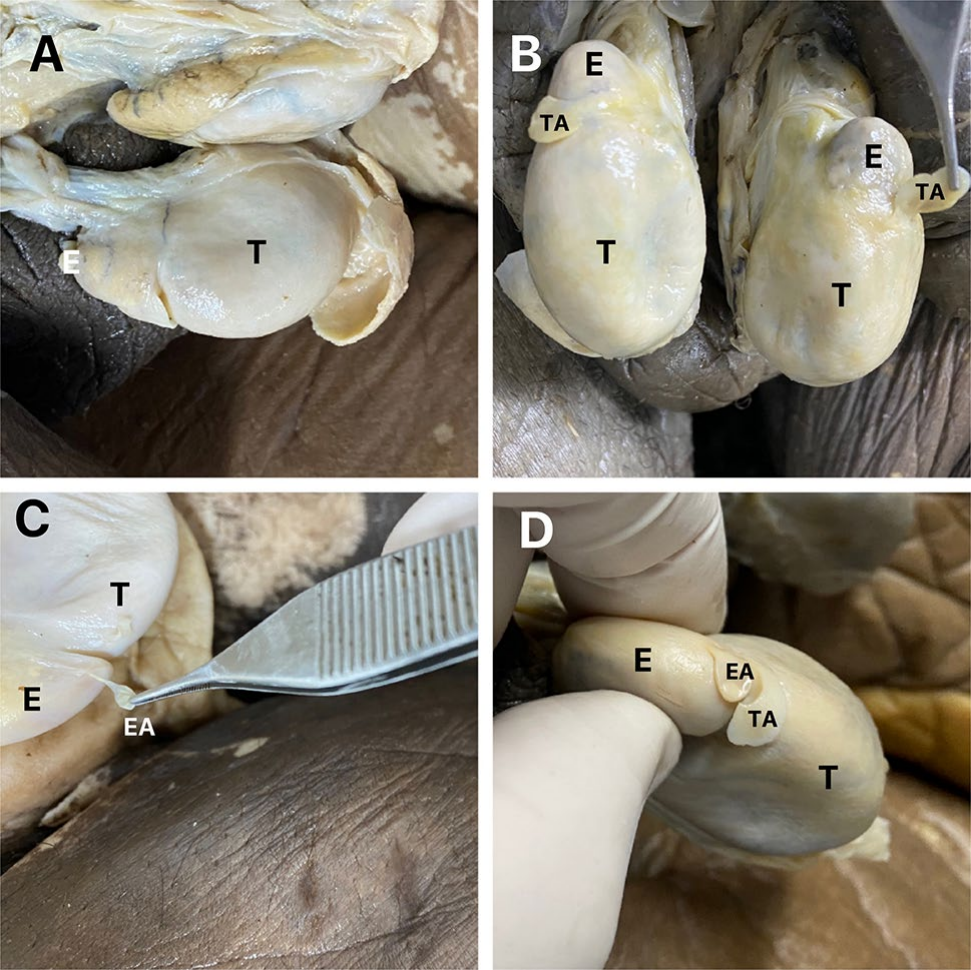
The figure shows the types of possible dispositions of the para-testicular apppendix found during the dissections in our sample: (**A**) absence of testicular and epididymis appendix, (**B**) presence of testicular appendix (TA), (**C**) presence of epididymal appendix (EA) and (**D**) presence of testicular appendix and epidydimal appendix; T-Testis and E-Epididymis.

The type and number of the testicular and epididymal appendices are shown in Table 1. In Fig. 3 we can observe the types of appendices found during our dissections.

**Table 1:** The table shows the type and laterality of appendages of our sample.

Type and laterality of appendages	Testicular appendage	Epididymal appendage	*p* value*
	n (%)	n (%)	
Absence of appendage	26 (34.2%)	49 (64.5%)	0.003
Unilateral pedicled appendage	11 (14.5%)	6 (7.9%)	0.302
Bilateral pedicled appendage	25 (32.9%)	14 (18.4%)	0.082
Unilateral sessile appendage	5 (6.6%)	1 (1.3%)	0.219
Bilateral sessile appendage	7 (9.2%)	5 (6.6%)	0.774
Bilateral + sessile pedicled appendage	0 (0.0%)	1 (1.3%)	1.000
Pedicled + sessile appendage	2 (2.6%)	0 (0.0%)	0.500
Total	76 (100%)	76 (100%)	

*McNemar Test.

The measurements of the testicles, testicular volume and appendices measurements are shown in Table 2.

**Table 2:** The table shows the mean and Standard deviaton (SD) of the measurements of the testis and appendagges of our sample.

Measurents	Right		Left	
	N	Mean ± SD	n	Mean ± SD
Testicle Length (Cm)	76	3.48 ± 0.53	76	3.34 ± 0.65
Testicle Width (Cm)	76	2.44 ± 0.44	76	2.32 ± 0.47
Testicle Thickness (Cm)	76	1.67 ± 0.52	76	1.64 ± 0.50
Testicle Volume	76	7.78 ± 3.51	76	7.12 ± 3.78
Testicular Appendage Length (mm)	45	3.20 ± 2.95	38	3.10 ± 3.75
Testicular Appendage Width (mm)	45	1.62 ± 1.58	38	1.55 ± 1.74
Testicular Appendage Thickness (mm)	45	0.38 ± 0.31	38	0.45 ± 0.51
Epididymal Appendage Length (mm)	26	3.70 ± 3.68	21	3.00 ± 3.11
Epididymal Appendage Width (mm)	26	1.90 ± 1.97	21	1.80 ± 2.10
Epididymal Appendage Thickness (mm)	26	0.47 ± 0.43	21	0.54 ± 0.56

In 50 cases (65.78%) we observed the presence of some type of hydatids and in 34 cases (44.7%) we observed the presence of testicular appendices with a significant association between the occurrence of these appendices on the right and left sides (*p* < 0.001). In 19 cases (25%) we observed the presence of epididymal appendices with a significant association between the occurrence of these appendices on the right and left sides (*p* < 0.001).

We observed the presence of pedicled appendices in 39 cases (51.32%), with 25 of the cases (32.89%) having pedicled testicular appendices (right testicular appendices mean length = 3.20 mm; left testicular appendices mean length = 3.01 mm) with a significant association between the occurrence of these pedicled appendices on the right and left sides (*p* < 0.001) and 14 of the cases (18.42%) were of pedicled epididymal appendices (right epididymary appendices mean length = 3.70 mm; left epididymal appendices mean length = 3.00 mm) with a significant association between the occurrence of pedicled epididymal appendices on the right and left sides (*p* < 0.001).

**Figure 4:**
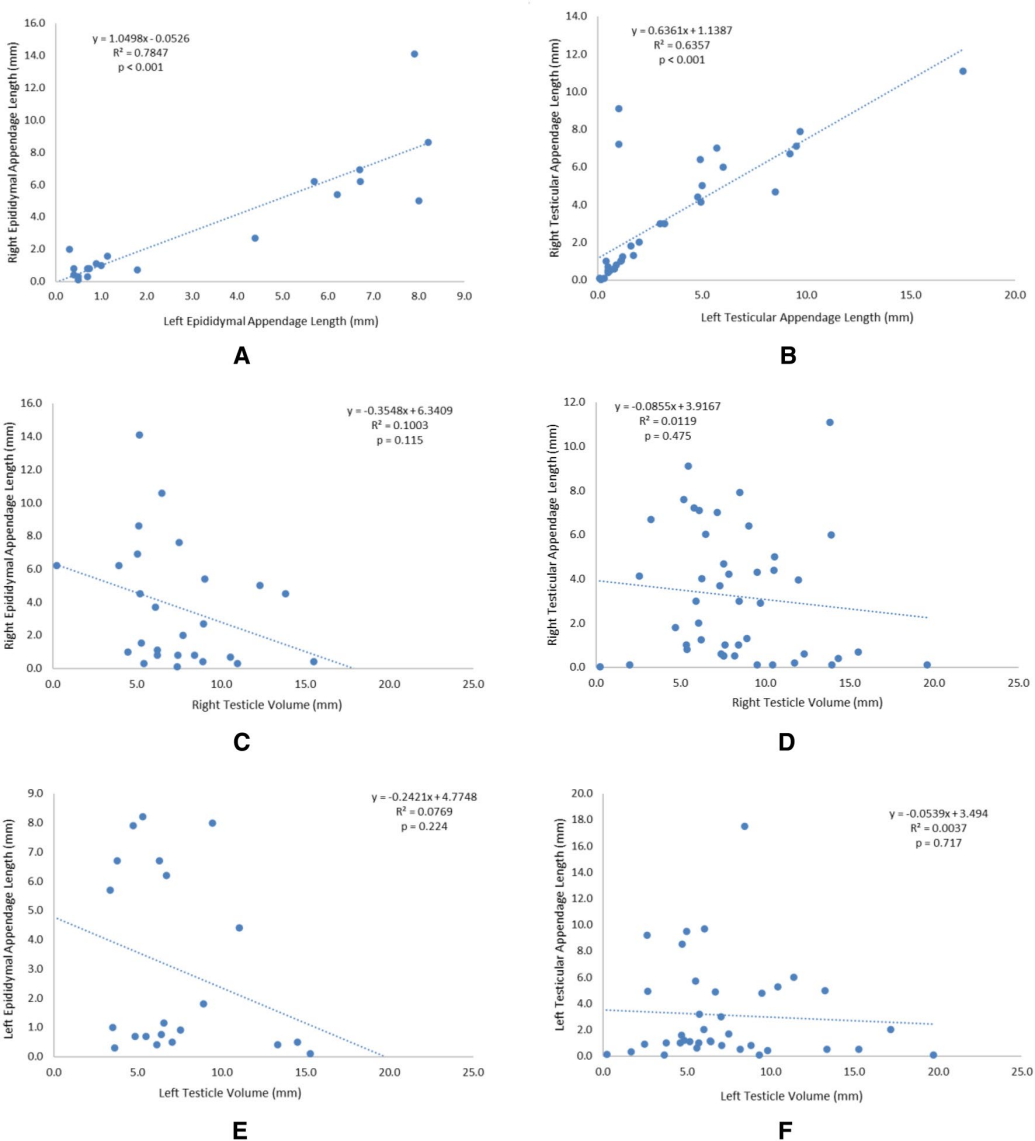
Linear regression analyses. (**A**) Right testicular apppendix lenght X left testicular appendix length; (**B**) Right epdidimary appendix length X left epididimary appendix length; (**C**) Right testicular appendix length X right testicular volume; (**D**) Left testicular appendix length X left testicular volume; (**E**) Right epididimary appendix length X Right testicular volume and (**F**) Left epididimary appendix length X left testicular volume.

The linear correlations comparing morphological data of testicular and epididimary appendices and testicular volume are reported in Fig. 4. The linear regression analysis indicated that the right testicular appendix lenght increased significantly and positively with left testicular appendix lenght (y = 0.6361x + 1.1387; r^2^ = 0,63,566), a moderate correlation and the right epdidimary appendices length increased significantly and positively with the left epididimary appendices length (y = 1.0498x − 0.0526 r^2^ = 0.78474), a strong correlation. The right testicular appendices length increased significantly and positively with right testicular volume (y = 0.0942x + 2.4195 r^2^ = 0.01474) a very weak correlation, and the left testicular appendices length increased significantly and positively with left testicular volume (y = 0.1408x + 2.0803 r^2^ = 0.02081), also a very weak correlation. The linear regression analysis indicated that the right epididimary appendices length increased significantly and positively with right testicular volume (y = − 0.0123x + 3.8007; r^2^ = 0.00019), a very weak correlation and the left epididimary appendices length increased significantly and positively with left testicular volume (y = − 0.2103x + 4.5593; r^2^ = 0.06833), also a very weak correlation (Supplementary file S1).

## Discussion

The testicular appendix is derived from the upper portion of the paramesonephric duct, also known as Morgagni’s sessile hydatid and is located at the upper end of the testis^21^. The epididymal appendix originates from the mesonephric duct, generally located in the head of the epididymis also known as Morgagni’s pedunculated hydatid^21^. Other vestigial structures derived from the mesonephric duct are the “Haller's organs”, located in the fissure between the testis and the epididymis, consisting of a group of superior and inferior aberrant vessels, and the “Giraldes’ organ”, called paradidymus or innominate body, and located in the distal portion of the spermatic cord^21,22^. Haller's organs and Giraldes' organ were not found in our sample.

Torsion of the testicular appendix is one of the most common causes of acute scrotum in the pediatric population^23^. This entity contributes to 31–67% of cases of acute scrotum and has a higher incidence than testicular torsion^23^. Torsion of the testicular appendix produces intense painful complaints but can often be located in a small juxta-testicular mass, which, when transilluminated, appears as a small dark spot (blue dot sign)^23,24^.

Hydatids torsion occurs when the appendix rotates along the axis of its pedicle. Because they are pedicled structures, they are more predisposed to torsion^23–25^. Torsion of the Hydatid of Morgagni contributes to 91% to 95% of all appendix torsions^9,23–25^ and occurs mainly in prepubertal age, between 7 and 14 years^9,23,25^. This clinical entity is more common on the left^9,23^. Torsion of the appendix can manifest itself as focal scrotal pain, with variable evolution time, which may persist for a week or more^23^. The progressive onset of pain may suggest the diagnosis of appendix torsion, as opposed to testicular torsion, which generally starts suddenly.

The treatment of this clinical entity is essentially conservative, with rest, ice, testicular support, and elevation and non-steroidal anti-inflammatory drugs^9,23^. Knowledge of the clinical findings and the typical ultrasound translation of torsion of the testicular appendix is essential to make the correct diagnosis and guide appropriate therapy, since torsion of the testicular appendix and epididymorchitis require conservative therapy and testicular torsion is a surgical emergency^9,26,27^.

Knowledge of the incidence, bilaterality and anatomical characteristics of the appendices, especially whether they are sessile or pedicled, is of great importance for indicating exploration of the opposite side during surgery to correct appendicular torsion.

An important previous study showed that the majority (90.4%) of emergency scrotal explorations in patients with torsion of the testicular appendix were unilateral^13^. In this study, the authors demonstrated that exploration of the contralateral side allows excision of appendices to achieve prophylaxis against future torsion of the testicular appendices and, probably, also torsion of the spermatic cord, reducing the occurrence of future episodes of testicular pain requiring access to an emergency service^13^.

In our sample we observed that in more than 30% of the cases the presence of pedicled appendices was bilateral. Our study showed that there is a tendency for bilateral pedicled testicular and epididymal appendices to occur. Of the cases studied, 45% have bilateral testicular appendices and 32% are bilaterally pedunculated. On the other hand, in 65% of the cases we do not observe epididimary appendices, but when present in 18%, they are pedicled bilaterally.

Our study, for the first time, in addition to evaluating the incidence of testicular hydatids, also evaluated and measured the size of the appendices, which would be a factor of great importance for the occurrence of torsion and would justify bilateral exploration.

Some limitations of this study should be mentioned: (a) the small sample size, and (b) the ideal sample for this study would be individuals in the age group where appendix torsions occur most frequently.

Testicular hydatids were present in around two thirds of our sample being pedunculated in almost half of the cases with bilateral similarity. There is a significant chance in cases of twisted appendices that the same anatomical characteristics are present on the opposite side, which is a factor that tends to indicate the need for contralateral surgical exploration in cases of torsion, although studies with larger samples are needed to confirm these findings.

### Supplementary Information

Supplementary Information.

## Abbreviations

TT: Testicular torsion
TV: Testicular volume
