# Supplementary material for: Morphometric study applied to testicular and epididymis hydatids torsion
Source: Sci Rep. 2024 Feb 8;14:3249. doi: 10.1038/s41598-024-52734-9 (PMC10853171; doi:10.1038/s41598-024-52734-9)
Supplement: Supplementary file 1 — Supplementary Information. [file 41598_2024_52734_MOESM1_ESM.pdf]

Dados transformados para Word

num	New data	initials	type	Corpse or Patient	Age (Years)	Weight (G)
1	0		Corpse	1		60000
2	0		Corpse	1		70000
3	0		Corpse	1		65000
4	0		Corpse	1		58000
5	0		Corpse	1		72000
6	0		Corpse	1		68000
7	0		Corpse	1		75000
8	0		Corpse	1		65000
9	0		Corpse	1		90000
10	0		Corpse	1		72000
11	0		Corpse	1		59000
12	0		Corpse	1		62000
13	0		Corpse	1		70000
14	0		Corpse	1		78000
15	0		Corpse	1		100000
16	0		Corpse	1		66000
17	0		Corpse	1		60000
18	0		Corpse	1		82000
19	0		Corpse	1		78000
20	0		Corpse	1		67000
21	0		Corpse	1		58000
22	0		Corpse	1		62000
23	0		Corpse	1		58000
24	0		Corpse	1		56000
25	0		Corpse	1		68000
26	0		Corpse	1		78000
27	0		Corpse	1		70000
29	0		Corpse	1		51000
30	0		Corpse	1		52000
31	0		Corpse	1		68000
32	0		Corpse	1		49000
33	0		Corpse	1		60000
34	0		Corpse	1		
35	0		Corpse	1		65000
36	0		Corpse	1		68000
37	0		Corpse	1		60000
38	0		Corpse	1		68000
39	0		Corpse	1		70000

40	0	Corpse	1		62000
41	0	Corpse	1		59000
42	0 J.B.	Patient	2	66	70000
43	0 R.P.B.	Patient	2	66	70000
44	0 V.P.	Patient	2	54	77000
45	0 J.E.	Patient	2	53	85000
46	0 E.F.A.	Patient	2	75	80000
47	0 P.C.B.P.	Patient	2	73	98000
48	0 W.S.	Patient	2	73	88000
49	0 G.K.	Patient	2	65	64000
50	0	Corpse	1		77000
51	0	Corpse	1		60000
52	0	Corpse	1		72000
53	0	Corpse	1		68000
45	1	Corpse	1		56000
46	1	Corpse	1		90000
47	1	Corpse	1		82000
48	1	Corpse	1		82000
49	1	Corpse	1		79000
50	1	Corpse	1		62000
51	1	Corpse	1		80000
52	1	Corpse	1		70000
53	1	Corpse	1		72000
54	1	Corpse	1		75000
55	1	Corpse	1		58000
56	1	Corpse	1		62000
57	1	Corpse	1		72000
58	1	Corpse	1		66000
59	1	Corpse	1		68000
60	1	Corpse	1		70000
61	1	Corpse	1		69000
62	1	Corpse	1		65000
63	1	Patient	2	72	72000
64	1	Patient	2	70	85000
65	1	Patient	2	70	78000
66	1	Patient	2	68	80000
67	1	Patient	2	80	67000
68	1	Patient	2	80	69000

Height (Cm)	Mensurements of The rigth testicle (Cm)	Lenght (Cm)	Width (Cm)	Thickness (Cm)
150	3.3x2.2x1.6	3.3	2.2	1.6
159	3.6x2.8x1.0	3.6	2.8	1
163	3.7x2.7x2.0	3.7	2.7	2
160	2.3x1.5x1.1	2.3	1.5	1.1
165	3.78x2.94x1.45	3.78	2.94	1.45
154	3.74x2.46x1.63	3.74	2.46	1.63
168	3.68x2.68x1.75	3.68	2.68	1.75
169	2.97x2.72x1.44	2.97	2.72	1.44
161	4.26x3.51x1.41	4.26	3.51	1.41
165	3.77x2.42x1.50	3.77	2.42	1.5
160	2.28x1.71x0.12	2.28	1.71	0.12
155	2.76x2.29x1.54	2.76	2.29	1.54
155	3.89x2.39x1.84	3.89	2.39	1.84
158	3.28x1.81x1.05	3.28	1.81	1.05
182	4.32x2.69x1.97	4.32	2.69	1.97
158	3.01x1.99x1.60	3.01	1.99	1.6
156	3.63x2.16x1.26	3.63	2.16	1.26
160	3.20x2.21x1.47	3.2	2.21	1.47
166	3.99x2.23x1.61	3.99	2.23	1.61
152	3.03x1.60x1.58	3.03	1.6	1.58
151	2.73x2.12x1x91	2.73	2.12	1.91
162	3.42x2.36x2.01	3.42	2.36	2.01
158	3.72x2.40x2.96	3.72	2.4	2.96
157	2.68x2.33x1.20	2.68	2.33	1.2
161	4.12x2.34x1.45	4.12	2.34	1.45
168	4.35x2.55x1.81	4.35	2.55	1.81
173	3.08x2.34x1.66	3.08	2.34	1.66
149	3.50x2.20x1.10	3.5	2.2	1.1
150	3.28x2.21x1.28	3.28	2.21	1.28
176	3.69x2.88x1.75	3.69	2.88	1.75
150	3.34x2.04x1.44	3.34	2.04	1.44
158	3.72x2.97x2.13	3.72	2.97	2.13
	3.31x1.74x1.47	3.31	1.74	1.47
172	3.96x3.43x2.18	3.96	3.43	2.18
176	3.33x2.23x1.96	3.33	2.23	1.96
167	3.93x2.70x1.64	3.93	2.7	1.64
165	2.00x1.70x0.13	2	1.7	0.13
183	3.02x2.64x1.81	3.02	2.64	1.81
171	3.50x2.21x1.33	3.5	2.21	1.33
170	4.20x2.66x1.29	4.2	2.66	1.29
160	4.10x2.80x1.50	4.1	2.8	1.5
172	4.10x2.60x2.50	4.1	2.6	2.5

180	3.50x3.00x2.00	3.5	3	2
179	3,2x2,2x1,6	3.2	2.2	1.6
163	3.00x2.00x2.70	3	2	2.7
180	4.80x3.0x2.6	4.8	3	2.6
173	4.00x2.10x2.40	4	2.1	2.4
180	3.70x2.10x1.90	3.7	2.1	1.9
158	4,21x3,16x0,93	4.21	3.16	0.93
162	3.14x1.91x0.82	3.14	1.91	0.82
169	3.57x2.58x1.98	3.57	2.58	1.98
168	3.70x2.87x1.36	3.7	2.87	1.36
163	3.40x2.20x1.90	3.4	2.2	1.9
182	3.50x2.50x2.00	3.5	2.5	2
176	3.80x2.50x2.10	3.8	2.5	2.1
179	3.20x2.60x1.70	3.2	2.6	1.7
171	4.00x3.00x1.60	4	3	1.6
155	3.50x2.00x1.70	3.5	2	1.7
192	3.40x2.30x2.00	3.4	2.3	2
180	3.70x2.80x1.00	3.7	2.8	1
173	3.90x2.70x2.60	3.9	2.7	2.6
170	4.50x3.00x1.50	4.5	3	1.5
168	2.70x2.50x2.70	2.7	2.5	2.7
159	3.90x2,50x2.30	3.9	2.5	2.3
177	3.10x2.20x1.50	3.1	2.2	1.5
162	3.10x2.40x1.60	3.1	2.4	1.6
162	3.00x2.30x1.30	3	2.3	1.3
169	2.90x2.10x1.40	2.9	2.1	1.4
170	3.20x2.20x1.50	3.2	2.2	1.5
165				
172	2.80x1.50x1.50	2.8	1.5	1.5
178	3.80x2.80x2.50	3.8	2.8	2.5
176	3.50x2.80x2.40	3.5	2.8	2.4
171	2.70x3.50x1.80	2.7	3.5	1.8
171	3.20x3.00x2.10	3.2	3	2.1
177	3.70x2.50x1.10	3.7	2.5	1.1

Mensurements of the left testicle (Cm)	Length (Cm)	Width (Cm)	Thickness(Cm)	Mensurements of the rigth testicular appendix (cm)
4.15x2.3x1.2	4.15	2.3	1.2	D: 2.0x1.0x0.5
3.2x2.3x1.7	3.2	2.3	1.7	
4.0x2.2x1.7	4	2.2	1.7	
2.4x1.2x1.1	2.4	1.2	1.1	D:0.10x0.10x0.05
2.85x2.90x1.48	2.85	2.9	1.48	D:1.02x0.59x0.18
2.24x1.60x0.89	2.24	1.6	0.89	D:4.21x3.51x0.74
3.28x2.40x1.63	3.28	2.4	1.63	D:6.39x3.50x0.56
3.05x2.58x1.21	3.05	2.58	1.21	D:7.1x4.4x0.8
3.72x2.94x1.82	3.72	2.94	1.82	
3.76x2.44x1.15	3.76	2.44	1.15	D:7.0x2.5x0.6
2.40x2.34x1.28	2.4	2.34	1.28	
2.60x2.33x1.67	2.6	2.33	1.67	
4.61x2.44x1.88	4.61	2.44	1.88	
3.0x2.1x0.8	3	2.1	0.8	D:6.7x3.8x1.1
3.68x2.12x1.67	3.68	2.12	1.67	D:3.96x2.98x0.80
3.33x1.99x1.81	3.33	1.99	1.81	
2.98x2.44x1.54	2.98	2.44	1.54	D:7.59x3.40x0.51
3.14x1.99x1.75	3.14	1.99	1.75	D:9.1x2.5x0.6
2.72x1.92x1.74	2.72	1.92	1.74	
2.74x2.23x1.69	2.74	2.23	1.69	
2.66x1.91x1.42	2.66	1.91	1.42	D:7.2x4.9x0.6
2.79x2.13x1.95	2.79	2.13	1.95	D:7.9x6.6x0.3
3.65x2.49x1.78	3.65	2.49	1.78	D:11.1x2.6x0.6
2.95x2.28x0.95	2.95	2.28	0.95	
3.53x2.47x1.62	3.53	2.47	1.62	D:3.7x2.1x0.4
4.21x2.71x1.59	4.21	2.71	1.59	D:4.4x2.3x0.3
3.54x2.01x1.28	3.54	2.01	1.28	D:4.0x2.3x0.2
Fora da bolsa				
Fora da bolsa				
3.09x1.95x1.26	3.09	1.95	1.26	D:2.9x1.0x0.7
2.96x2.42x1.26	2.96	2.42	1.26	
3.92x2.20x1.96	3.92	2.2	1.96	D:0.6x0.3x0.1
1.77x0.38x1.12	1.77	0.38	1.12	
4.10x2.92x2.13	4.1	2.92	2.13	D:0.7x0.6x0.4
3.90x2.90x1.66	3.9	2.9	1.66	D:1.0x0.4x0.1
2.84x2.32x1.82	2.84	2.32	1.82	
2.00x1.65x0.12	2	1.65	0.12	D:0.02x0.01
3.18x2.31x2.13	3.18	2.31	2.13	D:0.5x0.0.3x0.1
3.15x1.25x1.20	3.15	1.25	1.2	D:0.8x0.3x0.1
3.86x2.70x1.52	3.86	2.7	1.52	D:0.5x0.4x0.1
3.90x2.40x1.80	3.9	2.4	1.8	
4.50x2.30x2.40	4.5	2.3	2.4	D:0.10x0.10

3.70x3.00x2.50	3.7	3	2.5	
3.30x2.40x1.70	3.3	2.4	1.7	D:3.0x2.0x0.5
3.20x1.80x1.90	3.2	1.8	1.9	D:3.0x2.0x0.7
5.0x2.9x2.6	5	2.9	2.6	D:0.1x0.2x0.1
3.20x1.90x2.80	3.2	1.9	2.8	
2.90x2.70x2.10	2.9	2	1.2	
3,49x2,29x1,16	3.49	2.29	1.16	D:6.03x2.06x0.71
3.21x1.92x0.83	3.21	1.92	0.83	D:4.14x3.00x0.96
3.64x2.22x2.01	3.64	2.22	2.01	D:4,31x2,92x0,02
3.22x2.66x1.05	3.22	2.66	1.05	D:4.68x0.30x0.15
2.80x2.20x1.90	2.8	2.2	1.9	
3.60x2.70x2.30	3.6	2.7	2.3	
3.60x2.60x1.90	3.6	2.6	1.9	Dir: 0.1x0.1
3.00x2.50x1.80	3	2.5	1.8	Dir: 0.6x0.2
5.20x3.50x1.50	5.2	3.5	1.5	
3.30x2.30x1.90	3.3	2.3	1.9	
3.00x2.10x1.70	3	2.1	1.7	Dir: 0.5x0.1
3.50x2.30x1.30	3.5	2.3	1.3	
3.60x3.00x2.70	3.6	3	2.7	Dir: 0.40x0.20xx
4.20x2.80x1.30	4.2	2.8	1.3	
2.80x2.20x1.80	2.8	2.2	1.8	Dir: 0.10x0.05
3.60x2.70x2.20	3.6	2.7	2.2	Dir: 0.20x0.10
2.90x2.00x1.20	2.9	2	1.2	
3.00x2.20x1.40	3	2.2	1.4	Dir: 1.25x0.80
3.10x2.40x1.20	3.1	2.4	1.2	Dir: 1.80x1.50
2.80x2.00x1.20	2.8	2	1.2	
3.00x2.10x1.40	3	2.1	1.4	
3.00x2.20x1.50	3	2.2	1.5	
4.10x3.20x2.50	4.1	3.2	2.5	
3.10x2.60x2.70	3.1	2.6	2.7	Dir|: 6.00x3.00
4.30x2.10x2.00	4.3	2.1	2	
2.20x3.10x2.10	2.2	3.1	2.1	Dir: 1.30x0.10
43.40x2.80x2.10	4.3	2.8	2.1	Dir: 5.00x1.00
3.80x2.70x1.20	3.8	2.7	1.2	Dir: 1.00x0.60

Length (Cm)	Width (Cm)	Thickness(Cm)	Type do apêndice	Right apendage
2	1	0.5	2	1
				2
				2
0.1	0.1	0.05	1	1
1.02	0.59	0.18	1	1
4.21	3.51	0.74	1	1
6.39	3.5	0.56	1	1
7.1	4.4	0.8	1	1
				2
7	2.5	0.6	1	1
				2
				2
				2
6.7	3.8	1.1	1	1
3.96	2.98	0.8	2	1
				2
7.59	3.4	0.51	2	1
9.1	2.5	0.6	1	1
				2
				2
7.2	4.9	0.6	2	1
7.9	6.6	0.3	1	1
11.1	2.6	0.6	1	1
				2
3.7	2.1	0.4	1	1
4.4	2.3	0.3	1	1
4	2.3	0.2	1	1
				2
				2
2.9	1	0.7	2	1
				2
0.6	0.3	0.1	1	1
				2
0.7	0.6	0.4	2	1
1	0.4	0.1	1	1
				2
0.02	0.01	0.08	2	1
0.5	0.3	0.1	2	1
0.8	0.3	0.1	1	1
0.5	0.4	0.1	1	1
				2
0.1	0.1	0.1	1	1
				2

3	2	0.5	1	1
3	2	0.7	1	1
0.1	0.2	0.1	2	1
				2
			1	1
6.03	2.06	0.71	1	1
4.14	3	0.96	2	1
4.31	2.92	0.02	1	1
4.68	0.3	0.15	1	1
				2
				2
0.1	0.1	0.1	2	1
0.6	0.2	0.08	1	1
				2
				2
0.5	0.1	0.1	1	1
				2
0.4	0.2	0.1	1	1
				2
0.1	0.05	0.02	1	1
0.2	0.1	0.1	1	1
				2
1.25	0.8	0.2	1	1
1.8	1.5	0.8	1	1
				2
				2
				2
				2
6	3	1	1	1
				2
1.3	0.1	0.1	1	1
5	1	0.5	1	1
1	0.6	0.2	1	1

Right pedicle appendage	Mensurements of the left testicular appendages (cm)	Lenght (Cm)	Width (Cm)	Thickness (Cm)
2	E: 2.0x1.0x0.4	2	1	0.4
2				
2				
1	E:0.30x0.16x0.09	0.3	0.16	0.09
1	E:1.14x0.52x0.19	1.14	0.52	0.19
1	E:4.91x4.29x2.43	4.91	4.29	2.43
1	E:9.5x4.6x0.6	9.5	4.6	0.6
2	E:5.29x2.50x0.62	5.29	2.5	0.62
1	E:5.7x3.3x1.2	5.7	3.3	1.2
1	E:9.2x4.0x0.6	9.2	4	0.6
1	E:1.0x0.4x0.7	1	0.4	0.7
2	E:1.0x0.5x0.4	1	0.5	0.4
1	E:9.7x7.6x0.3	9.7	7.6	0.3
1	E:17.5x3.3x0.5	17.5	3.3	0.5
1	E:4.8x3.5x0.2	4.8	3.5	0.2
1	E:0.8x0.1x0.1	0.8	0.1	0.1
2	E:0.5x0.4x0.1	0.5	0.4	0.1
1	E:0.4x0.3x0.1	0.4	0.3	0.1
2	E:0.01x0.01	0.15	0.01	0.08
2	E:0.5x0.3x0.1	0.5	0.3	0.1
1	E:0.9x0.2x0.1	0.9	0.2	0.1

1	E:3.0x2.0x0.5	3	2	0.5
1	E:3.2x1.8x1.9	3.2	1.8	1.9
2	E:0.1x0.1x0.1	0.1	0.1	0.1
2				
1	E:0.1x0,1	0.1	0.1	0.1
2	E:4.94x1.59x0.49	4.94	1.59	0.49
1	E:8.52x4.14x0.77	8.52	4.14	0.77
2	Esq: 0.1x0.1	0.1	0.1	0.1
1	Esq: 0.8x0.3	0.8	0.3	0.1
1	Esq: 0.60x0.50	0.6	0.5	0.1
1	Esq: 0.50x0.30	0.5	0.3	0.1
1	Esq: 1.20x0.90	1.2	0.9	0.1
1	Esq: 1.60x1.20	1.6	1.2	0.8
2	Esq: 1.00x1.00	1	1	0.5
2	Esq: 1.10x0.80	1.1	0.8	0.2
2	Esq: 2.00x1.00	2	1	0.5
1	Esq: 6.00x2.25	6	2.25	0.9
1	Esq: 1.70x0.10	1.7	0.1	0.1
1	Esq: 5.00x3.00	5	3	0.9
1	Esq 1: 1.10x0.60	1.1	0.6	0.2

Type of the left appendages	Left appendages	Pedicle left appendages	Mensurements of the left appendages (2)	Length (Cm)
2	1	2		
	2	2		
	2	2		
1	1	1		
1	1	1		
	2	2		
1	1	1		
1	1	1		
1	1	1		
1	1	1		
	2	2		
	2	2		
	2	2		
1	1	1		
	2	2		
	2	2		
	2	2		
1	1	1		
	2	2		
	2	2		
2	1	2		
1	1	1		
1	1	1		
	2	2		
	2	2		
1	1	1		
	2	2		
	2	2		
	2	2		
	2	2		
	2	2		
1	1	1		
	2	2		
1	1	1		
1	1	1		
	2	2		
2	1	2		
2	1	2		
1	1	1		
	2	2		
	2	2		
	2	2		
	2	2		

1	1	1		
1	1	1		
2	1	2		
	2	2		
2	1	2		
	2	2		
2	1	2		
	2	2		
1	1	1		
	2	2		
	2	2		
2	1	2		
1	1	1		
	2	2		
	2	2		
1	1	1		
	2	2		
1	1	1		
	2	2		
	2	2		
	2	2		
	2	2		
1	1	1		
1	1	1		
	2	2		
2	1	2		
1	1	1		
2	1	2		
1	1	1		
	2	2		
1	1	1		
1	1	1		
1	1	1	Esq 2: 0.50x0.50	0.5

Width (Cm)	Thickness (Cm)	Typeof left testicle appendages (2)	Mensurements of the left epidydimal appendages (cm)	Length (cm)
			D: 1.55x0.8x0.4	1.55
			D:0.80x0.61x0.32	0.8
			D1:5.4x3.9x0.8	5.4
			D1:3.7X1.6X0.3	3.7
			D1:6.2x4.2x1.7	6.2
			D:8.62x7.76x1.17	8.62
			D:2.7x1.6x0.7	2.7
			D:6.92x3.58x0.88	6.92
			D:4.5x2.4x0.5	4.5
			D:7.6x2.7x0.5	7.6
			D:4.5x3.3x0.6	4.5
			D:6.2x4.0x0.8	6.2
			D:14.1x5.0x1.0	14.1
			D:0.4x0.2x0.1	0.4
			D:0.3x0.2x0.1	0.3

			D:0.7x0.1x0.1	0.7
			D 0.20x0.10x0.10	2
			D:10.56x2.90x0.2	10.56
			Dir: 0.8x0.3	0.8
			Dir: 0.1x0.1	0.1
			Dir 1: 1.10x0.30	1.1
			Dir: 0.30x0.20	0.3
			Dir: 0.80x0.70	0.8
			Dir: 1.00x0.50	1
			Dir: 5.00x2.00	5
			Dir: 0.40x0.40	0.4
0.5	0.33	1		

Width (Cm)	Thickness D1 (Cm)	Type of the right epidydimal appendage(cm)	Rigth epidydimal appendage	Right pedicle epidymal appendag
			2	2
0.8	0.4	2	1	2
			2	2
			2	2
0.61	0.32	2	1	2
			2	2
3.9	0.8	1	1	1
1.6	0.3	1	1	1
			2	2
			2	2
4.2	1.7	1	1	1
7.76	1.17	2	1	2
1.6	0.7	2	1	2
			2	2
			2	2
3.58	0.88	1	1	1
2.4	0.5	1	1	1
			2	2
2.7	0.5	1	1	1
			2	2
			2	2
			2	2
3.3	0.6	1	1	1
4	0.8	1	1	1
			2	2
			2	2
			2	2
			2	2
			2	2
			2	2
5	1	2	1	2
			2	2
			2	2
0.2	0.1	1	1	1
			2	2
			2	2
			2	2
			2	2
			2	2
			2	2
			2	2
			2	2

0.2	0.1	1	1	1
			2	2
			2	2
			2	2
0.1	0.1	1	1	1
0.1	0.1		2	2
2.9	0.2	1	1	1
			2	2
			2	2
			2	2
0.3	0.1	1	1	1
			2	2
			2	2
0.1	0.1	1	1	1
			2	2
0.3	0.08	1	1	1
			2	2
0.2	0.1	1	1	1
			2	2
			2	2
			2	2
			2	2
			2	2
0.7	0.1	1	1	1
			2	2
0.5	0.2	1	1	1
			2	2
			2	2
			2	2
			2	2
2	1	1	1	1
0.4	0.2	1	1	1
			2	2
			2	2

Mensurements of the right epidydimal appendages(cm)	Lenght (Cm)	Width (Cm)	Espessura do Apêndice Epididimário (cm)	Thickness (cm)
D2:3.6x2.7x0.9	3.6	2.7	0.9	2
D2:3.9X2.9X0.5	3.9	2.9	0.5	1
D2:5.5x5.0x1.0	5.5	5	1	1

Dir 2: 0.2x0.5	0.2	0.5	0.1	1

Mensurements of the left epidydimal appendage (cm)	Lenght (Cm)	Width (Cm)	Thickness (Cm)	Type of the left epidydimal appendage (cm)
E:1.15x0.79x0.39	1.15	0.79	0.39	2
E:0.75x0.42x0.13	0.75	0.42	0.13	2
E:6.2x3.5x1.8	6.2	3.5	1.8	1
E:6.71x3.04x1.32	6.71	3.04	1.32	1
E:8.2x7.9x1.1	8.2	7.9	1.1	2
E:4.4x3.3x1.2	4.4	3.3	1.2	2
E1:6.7x4.2x1.3	6.7	4.2	1.3	1
E:5.7x3.5x0.5	5.7	3.5	0.5	1
E:7.9x3.7x1.0	7.9	3.7	1	2
E:0.4x0.3x0.1	0.4	0.3	0.1	1
E:0.5x0.2x0.1	0.5	0.2	0.1	1

E:1.8x0.3x0.2	1.8	0.3	0.2	1
E:0.3x0.1	0.3	0.1	0.1	2
Esq: 0.4x0.1	0.4	0.1	0.1	1
Esq: 0.5x0.4	0.5	0.4	0.1	1
Esq 1: 0.9x0.3	0.9	0.3	0.1	1
Esq: 0.70x0.30	0.7	0.3	0.1	1
Esq: 0.10x0.10	0.1	0.1	0.1	1
Esq: 0.70x0.90	0.7	0.9	0.2	1
Esq: 1.00x0.50	1	0.5	0.2	1
Esq: 8.00x4.00	8	4	1.25	1

Left epidydimal appendage	Left pedicle epidydymal appendage (cm)	Mensurements(cm)	Lenght (cm)	Width (Cm)
2	2			
1	2			
2	2			
2	2			
1	2			
2	2			
1	1			
2	2			
2	2			
2	2			
1	1			
1	2			
1	2			
2	2			
2	2			
1	1	E2:3.3x2.5x1.1	3.3	2.5
2	2			
2	2			
2	2			
2	2			
2	2			
2	2			
2	2			
1	1			
2	2			
2	2			
2	2			
2	2			
2	2			
2	2			
1	2			
2	2			
2	2			
1	1			
2	2			
2	2			
2	2			
2	2			
2	2			
2	2			
2	2			
2	2			
1	1			

2	2
2	2
2	2
1	1
1	2
2	2
2	2
2	2
2	2
1	1
2	2
2	2
1	1
2	2
1	1
2	2
1	1
1	1
2	2
2	2
2	2
2	2
1	1
2	2
1	1
2	2
2	2
2	2
2	2
1	1
2	2
2	2
2	2

Thickness (Cm)	Type of the left epidydimal appendage (cm)	Mensurements of the vas aberrant haller (cm)	Type do apêndice vas aberrant haller (cm)	Mensurements of the vas aberrant haller (cm)
		1:3.3x2.9x1.4	1	2:3.0x2.0x1.3
1.1	1			

Type of the appendicle vas aberrant haller (2)	Number of the testicular appendages	Testicular appendages?	Type and laterality of the testicles appedages	Number of the epidydimal appendages
	2	1	5	0
	0	0	0	2
	0	0	0	0
	2	1	2	0
	2	1	2	2
	1	1	1	0
	2	1	2	3
	2	1	2	2
	1	1	1	0
	2	1	2	0
	0	0	0	3
1	0	0	0	2
	0	0	0	2
	2	1	2	0
	1	1	4	0
	0	0	0	3
	1	1	4	1
	2	1	2	0
	0	0	0	1
	0	0	0	0
	2	1	5	0
	2	1	2	0
	2	1	2	1
	0	0	0	2
	1	1	1	0
	2	1	2	0
	1	1	1	0
	0	0	0	0
	0	0	0	0
	1	1	4	0
	0	0	0	2
	2	1	2	0
	0	0	0	0
	2	1	3	2
	2	1	2	0
	0	0	0	0
	2	1	5	0
	2	1	5	0
	2	1	2	0
	1	1	1	0
	0	0	0	0
	1	1	1	0

0	0	0	2
2	1	2	0
2	1	2	0
2	1	5	0
0	0	0	2
2	1	3	1
1	1	1	1
2	1	5	0
1	1	1	0
2	1	2	0
0	0	0	2
0	0	0	0
2	1	5	0
2	1	2	2
0	0	0	0
0	0	0	3
2	1	2	0
0	0	0	2
2	1	2	1
0	0	0	0
1	1	1	0
1	1	1	0
0	0	0	0
2	1	2	2
2	1	2	0
0	0	0	2
1	1	4	0
1	1	1	0
1	1	4	0
2	1	2	0
0	0	0	2
2	1	2	1
2	1	2	0
3	1	2	0

Epidydimal appendages?	Type and laterality of the epidydimal appendages	Number of the vas aberrant haller	Total number of the appendages	Volume of the right testicle
0	0	0	2	6.0821
1	5	0	2	5.2779
0	0	0	0	10.4615
0	0	0	2	1.9871
1	5	0	4	8.4373
0	0	0	1	7.8522
1	6	0	5	9.0369
1	2	0	4	6.091
0	0	0	1	11.0391
0	0	0	2	7.1655
1	2	0	3	0.245
1	5	2	4	5.0964
1	5	0	2	8.957
0	0	0	2	3.2639
0	0	0	1	11.9867
1	2	0	3	5.0181
1	1	0	2	5.1728
0	0	0	2	5.4432
1	1	0	1	7.5007
0	0	0	0	4.0107
0	0	0	2	5.788
0	0	0	2	8.4944
1	1	0	3	13.8371
1	2	0	2	3.9235
0	0	0	1	7.3195
0	0	0	2	10.5125
0	0	0	1	6.2643
0	0	0	0	4.4349
0	0	0	0	4.8582
0	0	0	1	9.7377
1	5	0	2	5.1373
0	0	0	2	12.3219
0	0	0	0	4.433
1	2	0	4	15.504
0	0	0	2	7.6209
0	0	0	0	9.1117
0	0	0	2	0.2314
0	0	0	2	7.5559
0	0	0	2	5.3865
0	0	0	1	7.546
0	0	0	0	9.0164
0	0	0	1	13.9539
1	2	0	2	10.9956

0	0	0	2	7.4414
0	0	0	2	5.8978
0	0	0	2	8.4823
1	2	0	2	9.163
1	4	0	3	10.4458
1	1	0	2	19.6035
0	0	0	2	10.5558
0	0	0	1	7.4058
0	0	0	2	10.0531
1	2	0	2	7.7299
0	0	0	0	6.2308
0	0	0	2	6.4781
1	2	0	4	2.575
0	0	0	0	8.1891
1	2	0	3	9.5489
0	0	0	2	5.4245
1	2	0	2	7.5617
1	1	0	3	14.3351
0	0	0	0	10.6029
0	0	0	1	9.5426
0	0	0	1	11.7417
0	0	0	0	5.3564
1	2	0	4	6.2329
0	0	0	2	4.6967
1	2	0	2	4.4642
0	0	0	1	5.5292
0	0	0	1	
0	0	0	1	3.2987
0	0	0	2	13.9277
1	2	0	2	12.315
1	1	0	3	8.9064
0	0	0	2	10.5558
0	0	0	3	5.3276

Volume of the left testicle
5.9973
6.5513
7.833
1.6588
6.4048
1.6702
6.7185
4.9854
10.4222
5.5243
3.7639
5.2972
11.0725
2.6389
6.8218
6.2802
5.8631
5.7256
4.7579
5.4068
3.7775
6.0676
8.4705
3.3456
7.3958
9.4983
4.7688
3.9752
4.7258
8.8504
0.3944
13.352
9.8304
6.2788
0.2073
8.1925
2.474
8.2946
8.8216
13.0062
14.5299

6.1282
7.0497
5.7303
11.7056
9.3117
19.7397
8.9137
7.0686
14.2942
3.6442
7.5508
4.8542
2.6784
5.6077
8.5045
5.4795
4.709
15.2681
8.0048
5.8057
11.1966
3.6442
4.8381
4.6747
3.5186
4.6181
5.1836
17.174
11.3946
9.4562
7.499
13.2387
6.4465
